# Innovative spectrophotometric methods for the analysis of terbinafine and ketoconazole in pharmaceutical preparations with compliance to greenness and blueness metrics

**DOI:** 10.1038/s41598-025-25067-4

**Published:** 2025-11-18

**Authors:** Engy Attaa Bakr, Abdallah M. Zeid, Shereen M. Shalan, Yasser El-Shabrawy, Aya Saad Radwan

**Affiliations:** 1 Pharmaceutical Analytical Chemistry Department, Faculty of Pharmacy, Mansoura University, Mansoura, 35516 Egypt; 2 Department of Pharmaceutical Chemistry, Faculty of Pharmacy, Horus University- Egypt, New Damietta, Egypt

**Keywords:** Terbinafine, Ketoconazole, Spectrophotometry, Greenness assessment, Blueness assessment, Chemistry, Analytical chemistry, Green chemistry

## Abstract

Five simple, sensitive, and eco-friendly spectrophotometric techniques were developed for solving the highly overlapped spectra of Terbinafine HCl and Ketoconazole in their combined tablet formulation, for the first time without the common excipients interfering. The methods included third derivative spectrophotometry (D^3^) (Method I), ratio spectra difference spectrophotometry (Method II), first derivative of ratio spectra (Method III), induced dual-wavelength (Method IV), and dual-wavelength resolution (Method V). The evaluation of the developed methods was based on correlation coefficients, relative standard deviations, and limits of detection and quantitation. Statistical tests, including the variance ratio F-test and Student t-test, showed no significant differences between the results obtained from the developed methods and those from the established reference methods. The techniques were efficiently applied to analyze the cited drugs in commercial tablet formulations with high % recoveries and low % RSD values. An assessment of the methods’ environmental impact, using the Analytical Eco-scale, the Green Analytical Procedure Index (GAPI), Analytical Greenness Approach (AGREE) and blue applicability grade index (BAGI) metrics demonstrated their sustainability. The developed spectrophotometric methods don’t require prior separation steps, large volumes of organic solvents, or sophisticated instruments; therefore, they are well-suited for routine analysis and quality control of the specified drugs in their dosage forms.

## Introduction

**Fig. 1:**
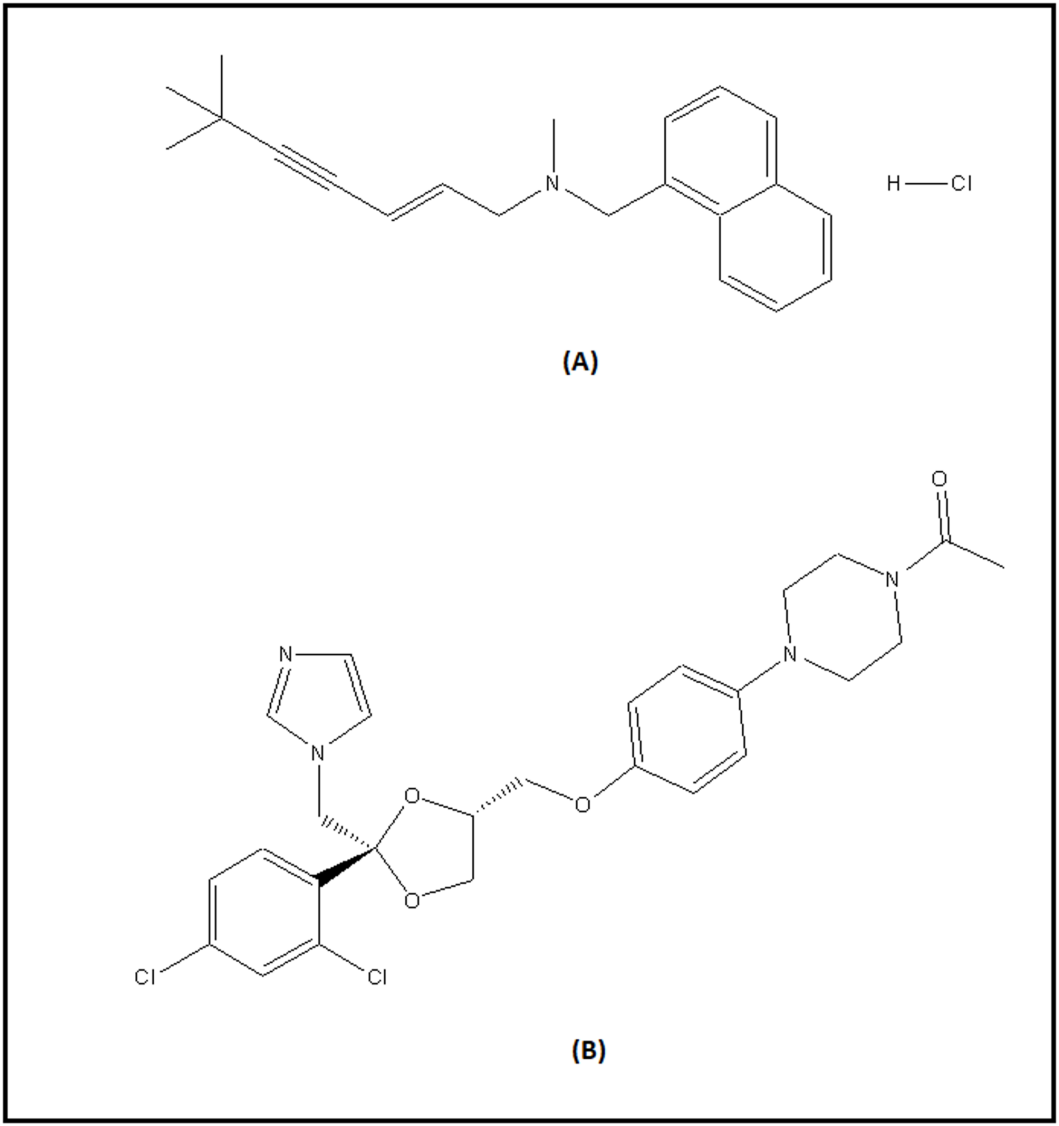
The chemical structures of Terbinafine HCL (**A**) and ketoconazole (**B**).

Terbinafine hydrochloride (TFH) **(**Fig. 1A**)** is an allylamine derivative with the chemical structure (2E)−6,6-dimethylhept-2-en-4-yn-1-yl(naphthalen-1-ylmethyl) amine hydrochloride^1^. Like other allylamines, TFH inhibits ergosterol synthesis by blocking the enzyme squalene epoxidase, which is essential in the fungal cell wall synthesis pathway^2^. In simpler terms, it disrupts the growth of fungal and bacterial cell walls, leading to cell death due to the loss of cellular protection. As a result, TFH is used topically to treat conditions such as dermatophytoses, pityriasis versicolor, cutaneous candidiasis^3^, and other superficial fungal infections like seborrheic dermatitis, tinea capitis, and onychomycosis, particularly due to its short-duration treatment regimen^4^.

Ketoconazole (KTZ) **(**Fig. 1B**)**, an imidazole derivative, is chemically identified as 1-acetyl-4-[4-[[(2RS,4SR)−2-(2,4-dichlorophenyl)−2-(1 H-imidazol-1-ylmethyl)−1,3-dioxolan-4-yl] methoxy] phenyl] iperazine^5^. This antifungal agent has both topical and systemic applications and can be formulated in various pharmaceutical forms, such as ketoconazole shampoo, which is effective for treating seborrheic dermatitis and pityriasis versicolor^6,7^. The primary action of imidazoles involves the inhibition of sterol-14α-desmethylase, an enzyme linked to cytochrome P450, leading to reduced fungal growth^8^. Together, TFH and ketoconazole are effective in treating a range of skin and nail fungal infections, including ringworm, and they have a broad-spectrum antifungal effect.

Numerous analytical techniques have been developed for determining TFH, including spectrophotometric^9–11^, spectrofluorimetric^12^, and chromatographic methods^13–16^.

KTZ was also analyzed using spectrophotometric^17–19^, spectrofluorimetric^20^ and chromatographic methods^21,22^. Meanwhile, only one chromatographic method has been published for the simultaneous determination of TFH and KTZ in a co-formulated dosage form^11^.

Compared to previously published methods, the new spectrophotometric approaches for the simultaneous determination of TFH and KTZ have numerous advantages, including simplicity, cost-effectiveness, eco-friendliness, and the absence of sophisticated instruments or prior separation. Furthermore, the majority of research labs have access to the spectrophotometric strategy, which is thought to be the most fundamental analytical technique. The proposed single-variable procedures successfully resolved the extreme spectral overlap between the two drugs and required fewer mathematical manipulations, giving the proposed methods distinct advantages over the described ones. This represents the first successful spectrophotometric application for determining these drugs in their tablet dosage form. The assay results obtained from these methods showed good agreement with those obtained from a reported HPLC method. Additionally, the procedures were validated in compliance with ICH criteria^23^. Also, a greenness assessment was successfully conducted using the Analytical Eco-scale, the Green Analytical Procedure Index (GAP), the Analytical Greenness Metric Approach (AGREE) and the blue applicability grade index (BAGI) metrics which revealed the excellent eco-friendliness of the developed methods. Accordingly, the suggested methods can be efficiently used for routine analysis of the co-formulated tablets of TFH and KTZ.

## Experimental

### Equipment

A Shimadzu ultraviolet–visible double beam (UV-1900I) Spectrophotometer (P/N 207–25700-58, Japan), along the spectrum wavelength range of 190–400 nm, using a monochromator with a Czerny-Turner mounting, 50 W Halogen Lamp, Deuterium Lamp, a spectral bandwidth of 1 nm and resolution of 1 nm was utilized for all measurements. Shimadzu analysis data system (LabSolutions DB/CS) enabled the manipulation of the integrated data.

The third derivative spectra, ratio and ratio derivative spectra for TFH and KTZ were derived using scaling factors = 10 and Δ λ = 8 nm.

### Reagents, materials, and solvents

Ethanol, methanol, and acetonitrile were sourced from Sigma-Aldrich in Germany. Hydrochloric acid and sodium hydroxide were obtained from El-Nasr Pharmaceutical Chemicals Co. in Egypt. Terbinafine hydrochloride (TFH) was generously provided by Novartis Pharma AG in Basel, Switzerland, with a purity of 99.2%. Ketoconazole (KTZ), labeled with a purity of 99.8%, was provided by Sigma-Aldrich.

### Standard stock solutions of TFH and KTZ

Stock standard solutions were prepared separately at a concentration of 1.0 mg/mL for each drug by dissolving 25.0 mg of TFH and KTZ with methanol, which were precisely measured in 25.0 mL volumetric flasks. After that, another dilution was performed with distilled water to create working solutions with a concentration of 100.0 µg/mL. The solutions, kept at 2 °C in a refrigerator, held their stability for at least seven days.

### Procedures

#### Construction of calibration graphs

Using distilled water as a blank, the zero-order absorption spectra of various solutions were recorded. A series of 10 mL volumetric flasks was filled with aliquots of the working solutions, diluted to the mark with distilled water, and mixed thoroughly to achieve final concentrations of TFH ranging from 0.6 to 12.0 µg/mL and KTZ from 1.0 to 10.0 µg/mL. After that, plotting the absorbance values versus the final concentrations of each drug(µg/mL) produces the calibration graphs from which the matching regression equations could be found.

##### Method (I): third derivative spectrophotometry (D^3^)

For TFH, the magnitudes of the third-order derivatives were recorded at 214.7 nm and at 208.6 nm for KTZ. Following that, the derivative spectrum intensities were plotted against the previously determined concentration ranges in order to create the calibration graphs and get the matching regression equations.

##### Method (II): ratio difference (RD)

KTZ divisor spectrum (3.0 µg/mL) divided the TFH spectra (0.6–12.0 µg/mL), and the divisor spectrum of KTZ (1.0–10.0 µg/mL) were divided by the spectra of TFH (4.0 µg/mL), then the variation in the TFH ratio spectrum amplitudes at 222.7 nm and 204.3 nm (∆P 222.7-204.3.7.3), whereas the KTH ratio spectra amplitude differences at 209.8 and 233.23 nm (∆P209.8–233.2.2), were plotted against the corresponding concentrations to provide the KTZ and TFH calibration curves, from which the appropriate regression equations were subsequently derived.

##### Method (III): first derivative of ratio spectrophotometric method (DD^1^)

After dividing the TFH (0.6–12.0 µg/mL) and KTZ (1.0–10.0 µg/mL) spectra by 3.0 µg/mL KTZ as a divisor and 4.0 µg/mL TFH as a divisor, respectively, the DD^1^ was calculated using a scaling factor = 10 and ∆λ = 10, then the DD^1^ amplitudes at 214.3 nm for TFH and 211.5 nm for KTZ, were plotted against the corresponding concentrations to provide the KTZ and TFH calibration curves, from which the appropriate regression equations were subsequently derived.

##### Method (IV): induced dual wavelength method (IDW)

As previously mentioned, various concentrations (1.5, 2.5, 3.75, 5.0, and 6.25 µg/mL) were made using the TFH ratio in dosage forms (1 KTZ: 2.5 TFH). At 222.7 nm and 231.3 nm, the absorbance was measured. The equality factor required to lessen the influence of KTZ in the combination can be found by calculating the absorbance of the equivalent KTZ concentrations at the precise wavelengths and splitting the initial absorbance measurement (222.7 nm) by the second one (231.3 nm). The absorbance of TFH at 231.3 nm was then multiplied by this equality factor, and the result was deducted from the absorbance at 222.7 nm to obtain ΔA. The calibration graph was created, and the regression equation was established by plotting ΔA against the required concentration.

##### Method V: dual wavelength resolution technique (DWR)

First, the drug’s absorption spectrum was divided by its matching concentration to create the normalized absorptivity curve for TFH. Absorptivity curves were produced as a result, and an average absorptivity curve was computed. The computed concentration of TFH was then multiplied by its median normalized absorption graph to obtain the spectrum of absorption of TFH. The computed TFH spectrum was then subtracted from the mixture’s total spectrum to determine the absorption spectra of KTZ. The first derivative was then computed (∆λ = 8 nm, scaling factor = 10). At 231.8 nm, the absorbance of KTZ was measured. Plotting the amplitudes at the selected wavelengths over the pertinent concentrations allowed for the creation of a calibration graph and the derivation of the regression equation.

#### Laboratory- prepared tablets

Since TFH and KTZ pills are not available in the Egyptian market, the dosage form was simulated using a premade combination. For the tablet formulation, 250.0 mg of TFH, 100.0 mg of KTZ HCl, along with starch, talc, gelatin, Avicel pH 112 FMC, and magnesium stearate were weighed. The constituents were mixed thoroughly in a porcelain mortar. After that, they moved to a 100.0 mL volumetric flask. About 60.0 milliliters of methanol were added, and the mixture was sonicated for 30 min before adjusting the volume to 100.0 mL with the same solvent. The solution was then double filtered through a 0.45 μm syringe filter. Aliquots were taken and moved to 10.0 mL measuring flasks, which were filled with distilled water to the mark for achieving concentration ratios of 2.5:1.0, 5.0:2.5, and 7.5:3.0 for TFH to KTZ, respectively.

## Results and discussion

**Fig. 2:**
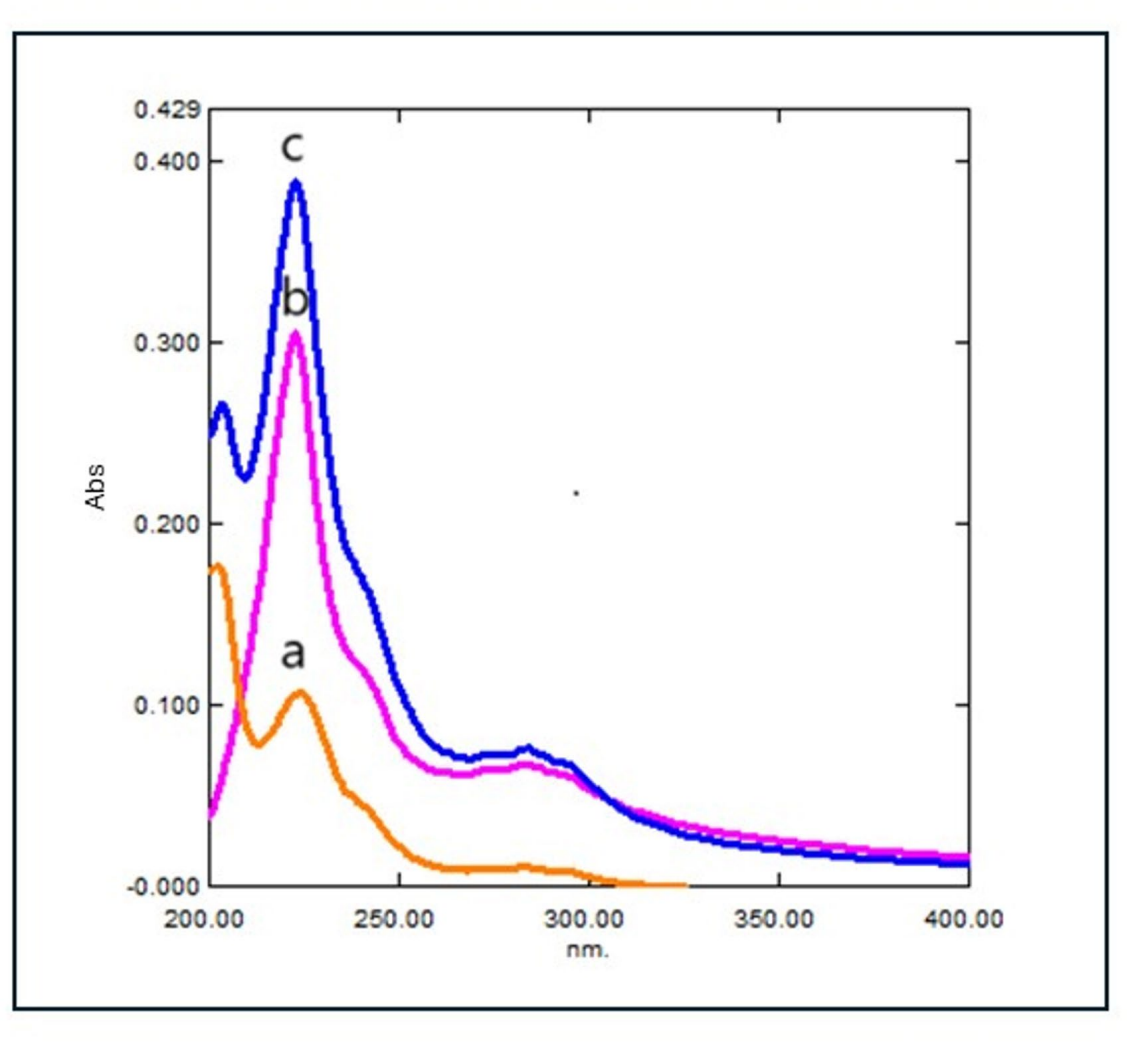
Zero order absorption spectra of KTZ (**a**; 1.0 µg/mL), TFH (**b**; 2.5 µg/mL), and mixture (**c**) in distilled water.

The overlap between TFH and KTZ’s UV spectrum (with the ratio of 2.5:1, TFH: KTZ) shows the extreme overlapping between the two drugs which hinders the direct determination of them (Fig. 2**)**. By adjusting UV spectra using several multi-component UV spectrophotometric approaches, this issue was resolved. This paper presents five straightforward, quick, and precise spectrophotometric techniques for the concurrent measurement of TFH and KTZ. These techniques comprised D^3^, RD, DD^1^, IDW and DWR.

### Method optimization

Various aspects influencing the suggested approaches’ performance were thoroughly examined.

#### Selection of solvent

For the examination of TFH and KTZ, solvents with various polarity values, including distilled water, methanol, pure ethanol, acetonitrile, in addition to an aqueous solvent with various pH values, such as pH 4 of acetate buffer and pH 8 of borate buffer, 0.1 M HCl, and 0.1 M NaOH, were investigated. To make the established procedures more environmentally friendly, distilled water was used to prepare TFH and KTZ solutions because it demonstrated superior solubility properties and absorption intensities for both.

#### Scaling factor and delta lambda for techniques

To maximize the performance of D^3^ derivatization, several scaling factors (SF) and delta lambda (∆λ) parameters were examined. The ideal values were discovered to be SF = 10 and ∆λ = 8 nm. Regarding ratio approaches, the selected values yielded the best results in terms of maximum intensity of absorption and least noise, especially at low concentrations.

### Features of the methods

#### Method I: D^3^ spectrophotometric method

**Fig. 3:**
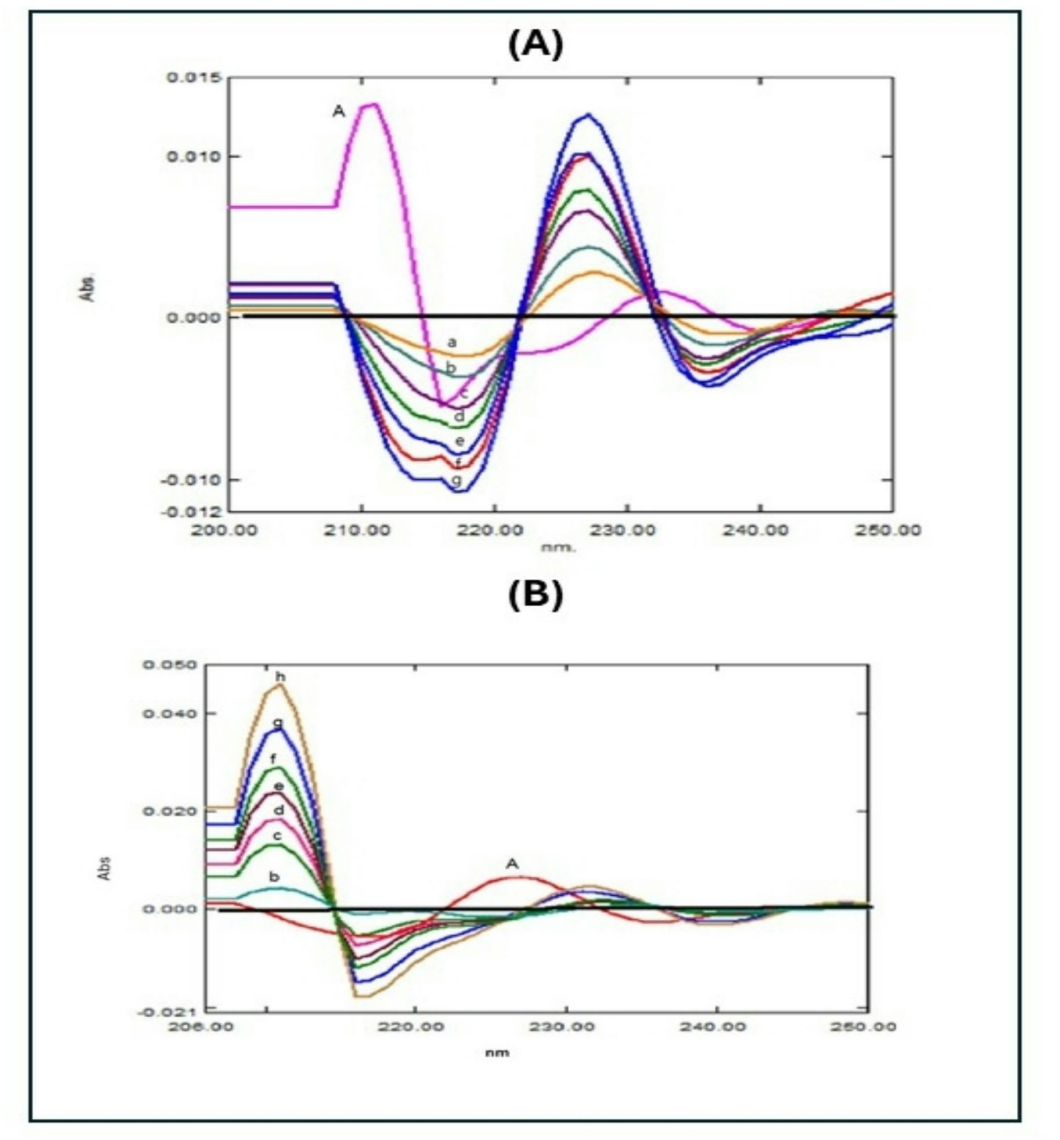
**(A)** Third derivative spectra of TFH (b-h: 0.6, 1.0, 4.0, 6.0, 8.0, 10.0, and 12.0 µg/mL) and a fixed concentration of KTZ (**a**) (3.0 µg/mL), **(B)**: Third derivative spectra of KTZ (b-h: 1.0, 3.0, 4.0, 5.0, 6.0, 8.0, and 10.0 µg/mL) and a fixed concentration of TFH (**a**) (1.0 µg/mL).

An analysis of TFH and KTZ utilizing zero-crossing point derivative spectrophotometry was performed. As seen in Fig. 3, the binary mixture zero-order UV spectra were converted to D^3^ utilizing SF = 10 nm and ∆λ = 8. For TFH and KTH measures, respectively, 214.7 nm and 208.6 nm were selected because they showed the highest D^3^ magnitude readings (in the absence of KTZ and TFH overlap). Table 1 shows the calibration curves’ statistical properties^24^.

#### Method II: RD spectrophotometric method

**Fig. 4:**
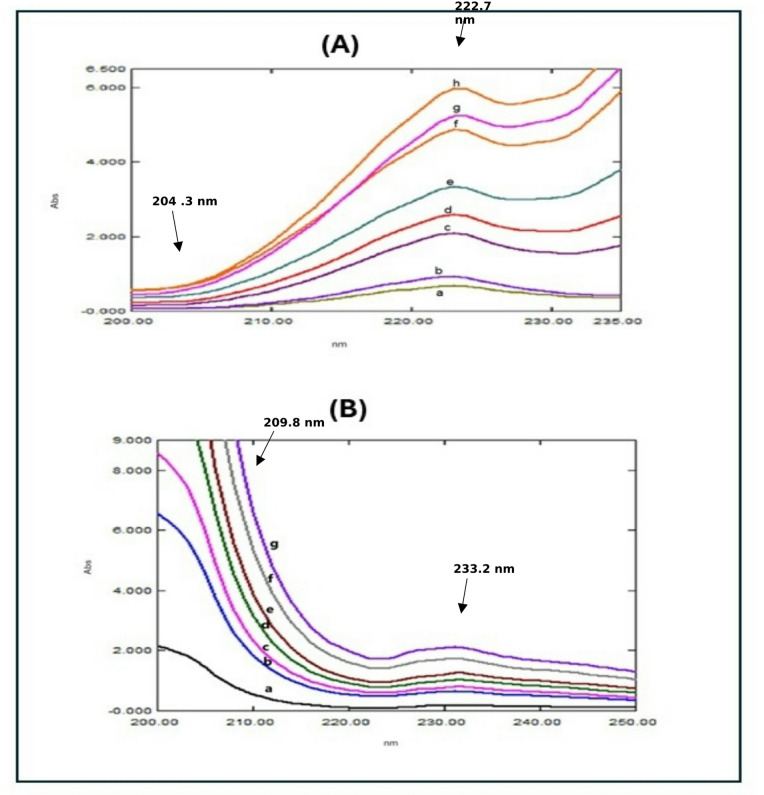
**(A)**: Ratio spectrophotometric spectra of different concentrations of TFH using 3.0 µg/mL KTZ as a divisor, where: (a-h) THF at concentrations of (0.6, 1.0, 3.0, 4.0, 6.0, 8.0,10.0 and 12.0 µg/mL) **(B)**: Ratio spectrophotometric spectra of different concentrations of KTZ using 4.0 µg/mL THF as a divisor, where: (a-g) KTZ at concentrations of (1.0, 3.0, 4.0, 5.0, 6.0, 8.0, and 10.0 µg/mL).

For enhancing the RD approach, two key steps were undertaken. First, the appropriate divisor concentration was determined. Different TFH concentrations were examined, and a divisor concentration of 3.0 µg/mL KTZ was identified as optimal for quantifying TFH in the prepared mixtures. Similarly, different concentrations of KTZ were evaluated, revealing that a divisor concentration of 4.0 µg/mL TFH was ideal for quantifying KTZ. The second step involved selecting the wavelength values for measurements. Two wavelengths were chosen to maximize the ratio spectrum’s absolute difference and guarantee strong linearity. For TFH determination, the amplitude difference between 222.7 nm and 204.3 nm (∆P222.7–204.3.3) was selected, while for KTZ determination, the amplitude difference between 209.8 nm and 233.2 nm (∆P209.8–233.2.2) was chosen **(**Fig. 4**)**, both provided satisfactory recovery percentages^25^.

#### Method III: DD^1^ spectrophotometric method

**Fig. 5:**
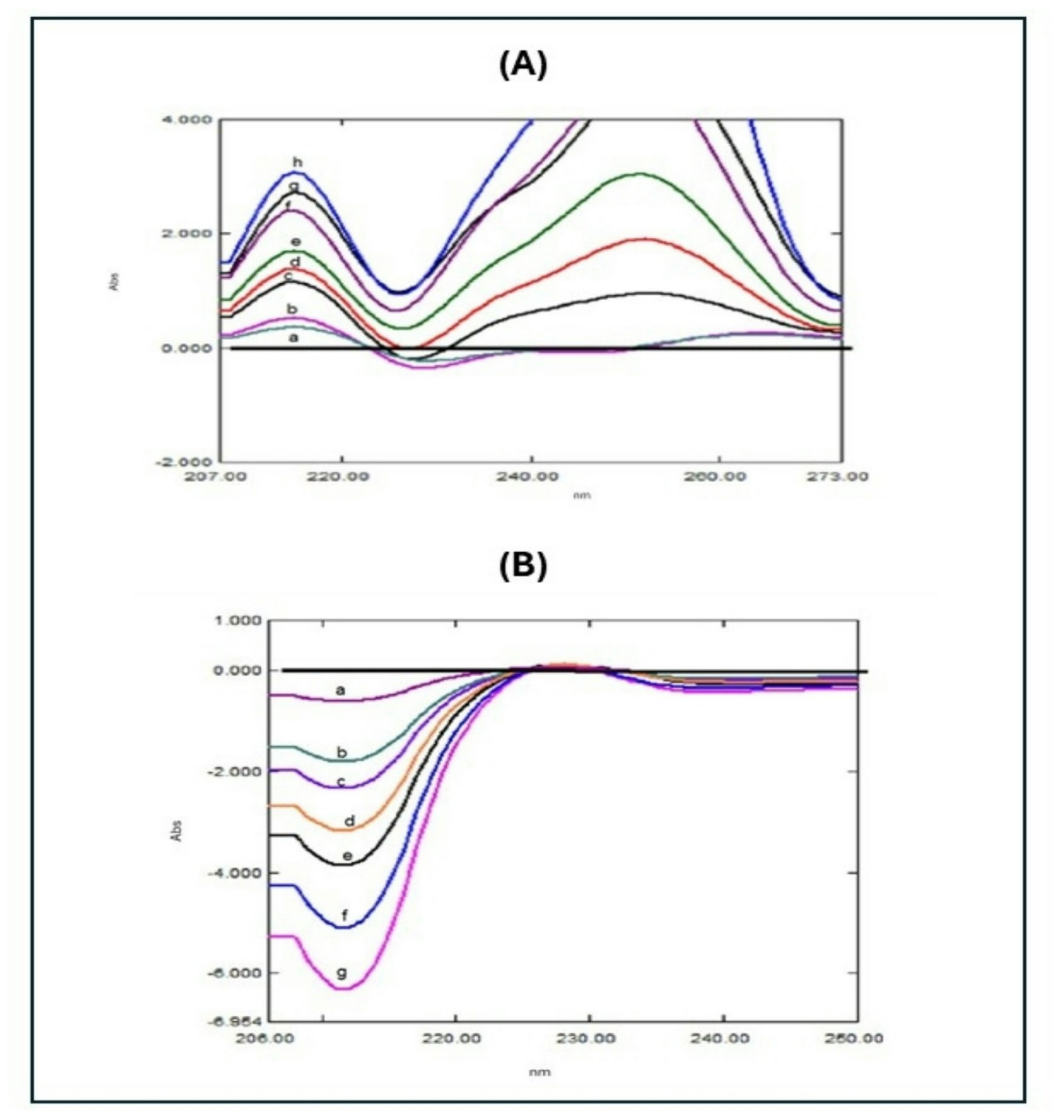
**(A)**: First derivative ratio spectrophotometric spectra of different concentrations of TFH (a-h: 0.6, 1.0, 3.0, 4.0, 6.0, 8.0, 10.0, and 12.0 µg/mL), **(B)**: First derivative ratio spectra of different KTZ concentrations (a-g: 1.0, 3.0, 4.0, 5.0, 6.0, 8.0, and 10.0 µg/mL).

As demonstrated in (Fig. 5**)**, the ratio spectrum was converted into a D^1^ utilizing ∆λ = 8 nm and SF = 10. For the quantitative assessment of TFH and KTZ in a co-formulated pill, the magnitude of DD^1^ of TFH was recorded at 214.3 nm, and the magnitude of KTZ was recorded at 211.5 nm^25^.

#### Method IV: induced dual wavelength method (IDW)

This approach is meant for usage with a mixture of (X and Y) that displays absorption spectra of zero order at two certain wavelengths, λ1 & λ2, that totally overlap. In this case, the absorbance difference is not zero since the absorbance of the interfering compounds at these certain wavelengths is not equivalent. The traditional dual-wavelength approach is inappropriate in this situation. The method is comparatively new and has only been used a few times to deal with complicated combinations of components^26^.

In summary, the following equations describe this circumstance:

1 $$A_{1} = {\text{ }}A_{{X1}} + {\text{ }}A_{{Y1}} at~~~\lambda 1$$

2 $$A_{2} = {\text{ }}A_{{X2}} + {\text{ }}A_{{Y2}} at~~~\lambda 2$$

Where:

A_1_ is the absorbance of the mixture at λ_1_, which is selected as the λ_max_ of X.

A_2_ is the absorbance of the mixture at λ_2_, which is another wavelength.

To remove component Y’s effect at the two wavelengths, an equality factor is computed as follows:

F_Y_ = A_Y1_/A_Y2_ so A_Y1_ = F_Y_A_Y2_.

Substitution in Eq. (1) gives:

3 $$A_{1} = {\text{ }}A_{{X1}} + {\text{ }}F_{Y} A_{{Y2}}$$

The equality factor FY is then multiplied by Eq. (2) to obtain:

4 $$F_{Y} A_{2} = {\text{ }}F_{Y} A_{{X2}} + {\text{ }}F_{Y} A_{{Y2}}$$

Equation (4) subtracted from Eq. (3) yields:

5 $$\Delta A{\text{ }}\left( {A_{1} - {\text{ }}F_{Y} A_{2} } \right){\text{ }} = {\text{ }}A_{{X1}} - {\text{ }}F_{Y} A_{{X2}}$$

The examination of Eq. (5) shows that the mixture’s absorbance difference is solely dependent on the CX, unaffected by CY. Therefore, the following regression equation can be used to calculate the concentration of component X:

6 $$\left( {\Delta A{\text{ }} = {\text{ }}A_{1} - {\text{ }}F_{Y} A_{2} } \right) = slope.C_{X} \pm intercept$$

The related regression equation can be constructed by graphing the absorbance difference values of the pure X zero-order spectra at the chosen wavelengths (ΔA = A_1_ − F_Y_A_2_) against the respective concentrations of X.

#### Method V: dual wavelength resolution technique (DWR)

**Fig. 6:**
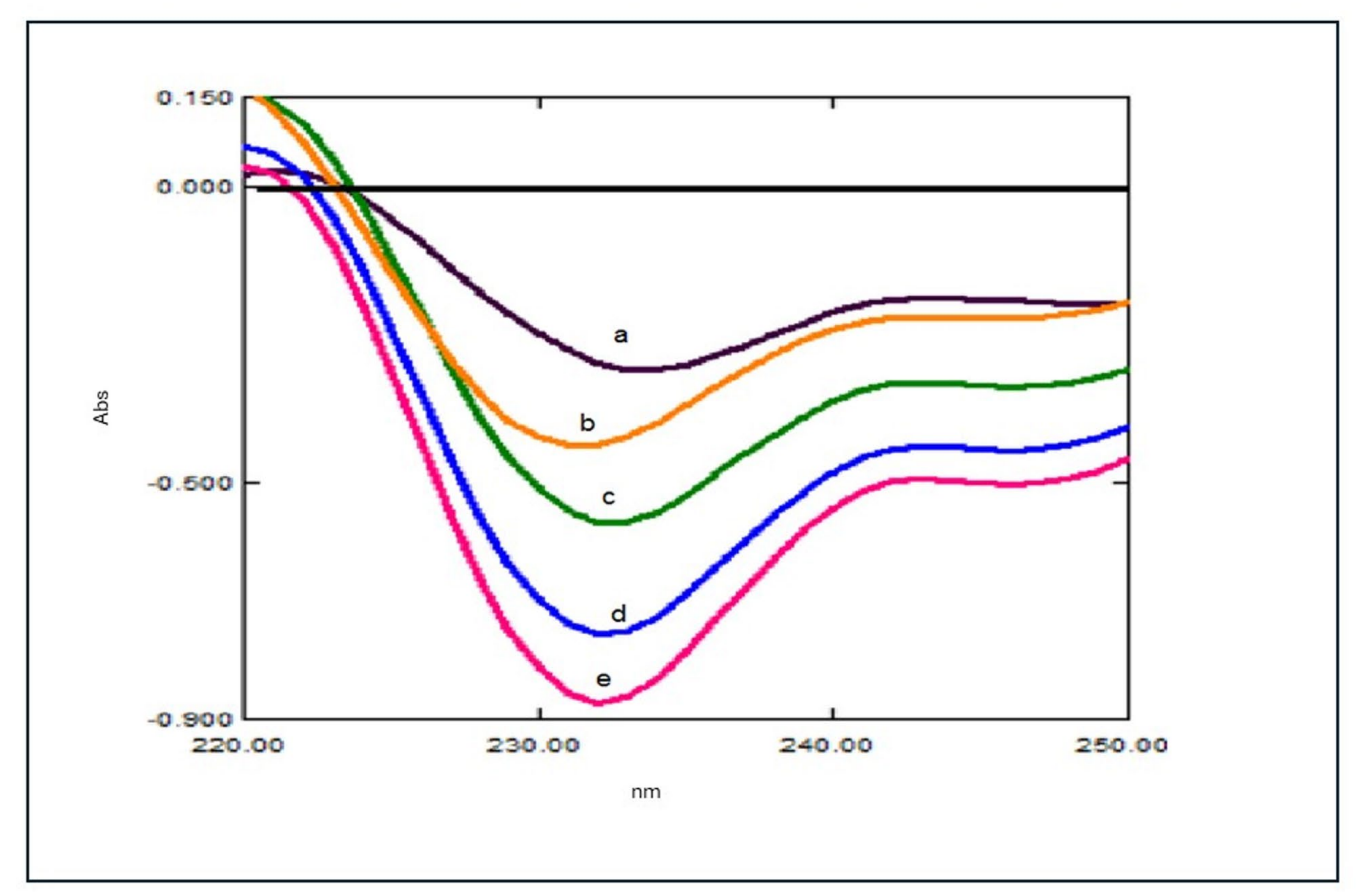
KTZ dual wavelength resolution at 231.8 nm (a-g: 0.6, 1.0, 1.5, 2.0, and 2.5 µg/mL).

When the concentration of the second component, Y, cannot be ascertained using the IDW approach, this technique is employed. The first component’s concentration, X, is ascertained using the IDW approach. Next, the calculated concentration is multiplied by component Y’s normalized absorptivity curve to obtain the zero-order spectrum of X. The complete spectrum of X is divided by its corresponding concentration to produce the normalized absorptivity curve of X, which displays the analyte’s (aX) absorptivity over all observed wavelengths. The spectra of component Y are then obtained by subtracting the computed spectrum of X from the mixture’s spectrum. Following the determination of the first derivative spectrum, the matching regression equation is carried out in order to address the issue of gaining indefinite peaks in Y’s zero-order spectrum^26^ Fig. 6.

### Validation of the suggested techniques

In accordance with the guidelines provided by ICH Q2 R1^23^, the suggested approaches were evaluated for a number of validation factors, including linearity, range, precision, accuracy, sensitivity, and selectivity.

#### Linearity and range

Calibration curves for TFH and KTZ were constructed using seven concentration levels within the ranges of 0.6–12.0 µg/mL for TFH and 1.0–10.0 µg/mL for KTZ. The obtained data demonstrated a strong linear relationship for both analytes. High correlation coefficients (r) and low standard deviations of residuals (Sy/x), intercepts (Sa), and slopes (Sb) confirmed the reliability of the regression models.

To provide a more rigorous assessment, additional statistical parameters were calculated. The relative standard deviation of the slope (Sb%) and the variance of the slope (Sb²) indicated excellent precision and minimal scatter of data points around the regression lines. The Student’s t-test was performed to verify that the intercepts were not significantly different from zero at the 95% confidence level, while the F-test confirmed the high significance of the regression models. Collectively, these results, summarized in Table 1, demonstrate the excellent linearity and robustness of the proposed methods^27,28^.

**Table 1:** Analytical performance data for determination of the TFH and KTZ by the proposed spectrophotometric methods.

Parameters	TFH				KTZ			
	D^3^	RD	DD^1^	IDW	D^3^	RD	DD^1^	DWR
Linearity (µg/mL)	0.6–12.0	0.6–12.0	0.6–12.0	1.5–6.25	1.0–10.0	1.0–10.0	1.0–10.0	0.6–2.5
LOD (µg/mL)	0.14	0.05	0.09	0.08	0.28	0.18	0.20	0.04
LOQ (µg/mL)	0.44	0.16	0.27	0.24	0.86	0.56	0.62	0.11
Correlation Coefficient (r)	0.9999	0.9999	0.9999	0.9999	0.9997	0.9998	0.9998	0.9999
Slope	9.74*10^−4^	0.51	0.45	0.16	3.19*10^−3^	0.51	0.64	0.32
Intercept	2.64*10^−4^	−0.15	5.35*10^−2^	−0.04	2.91*10^−4^	−0.15	−0.11	0.08
S_y_/x (standard deviation of residuals)	4.60*10^−5^	1.40*10^−2^	8.10*10^−3^	5.00*10^−3^	2.50*10^−4^	2.71*10^−2^	3.65*10^−2^	3.00*10^−3^
S_a_ (standard deviation of the intercept of the regression line)	1.46*10^−4^	9.82*10^−4^	5.51*10^−3^	2.00*10^−2^	2.73*10^−4^	7.79*10^−2^	9.78*10^−2^	3.41*10^−2^
S_b_ (standard deviation of the slope of the regression line)	1.87*10^−5^	9.59*10^−3^	7.40*10^−3^	3.16*10^−3^	3.36*10^−5^	9.59*10^−3^	1.2*10^−2^	6.59*10^−3^
Sb%	1.92	1.87	1.64	1.99	1.05	1.87	1.86	2.08
Sb^2^ (Variance of slop)	3.94* 10^−10^	9.19*10^−5^	5.48*10^−5^	9.98*10^−6^	1.13*10^−9^	9.19*10^−5^	1.45*10^−4^	4.35*10^−5^
t test	1.80	1.94	0.92	2.26	1.06	1.92	1.17	2.03
F-value	2.71*10^3^	2.86*10^3^	6.91*10^3^	2.52*10^3^	9.00*10^3^	2.86*10^3^	2.89*10^3^	2.29*10^3^
% Error	0.18	0.34	0.18	0.33	0.42	0.33	0.31	0.31
%RSD	0.48	0.91	0.49	0.74	1.04	0.83	0.77	0.69
Mean	99.71	99.74	100.13	100.06	99.48	99.91	99.94	99.92

#### Detection and quantitation limits

Using the following formulas, the limits of quantitation (LOQ) and limits of detection (LOD) for TFH and KTZ were determined in accordance with ICH guidelines.

*LOQ = 10 Sa/b *LOD = 3.3 Sa/b.

Where Sa = the standard deviation of the intercept of the calibration graph.

b = the slope of the calibration graph.

Table 1 summarizes the computed values, indicating the methods’ high sensitivity.

#### Accuracy and precision

The recommended methods for analyzing TFH and KTZ raw materials were carried out within the specified concentration values. The procedure’s accuracy was validated by the % good recovery percentages, as indicated in Table 2. The estimation results for the two medications were contrasted with the findings of an earlier HPLC chromatographic technique that employed phosphate buffer and acetonitrile as the mobile phase (40:60 v/v, pH 5.0) and employed PDA detection at 247 nm^11^. There were no significant variations in accuracy between the two approaches, according to the data in Table 2. Statistical investigation employing the student t-test and the variance ratio F-test, respectively, confirmed this^29^.

The suggested approaches’ intra-day precision was attained by measuring three distinct drug concentrations in the raw ingredients at three separate periods of time, and by analyzing these concentrations for three days in a row, the inter-day precision was assessed. Low percentages of RSD and %Error, as shown in Table 3, proved the precision of the proposed procedures.

**Table 2:** Application of the suggested spectrophotometric techniques for the determination of TFH and KTZ in raw materials.

Parameters	Drug	TFH									KTZ				
	Method	D^3^		RD		DD^1^		IDW			D^3^	RD	DD^1^	DWR	
		At 214.77 nm		At (222.77-204.31nm)		At 214.3 nm		At (222.77-231.38) nm			At (208.68) nm	At (209.85 - 233.23 nm)	At (211.5) nm	At (231.8) nm)	
		Taken conc. (µg/mL)	Found conc. (µg/mL)	Taken conc. (µg/mL)	Found conc. (µg/mL)	Taken conc. (µg/mL)	Found conc. (µg/mL)	Taken conc. (µg/mL)	Found conc. (µg/mL)	Taken conc. (µg/mL)	Found conc. (µg/mL)	Found conc. (µg/mL)	Found conc. (µg/mL)	Taken conc. (µg/mL)	Found conc. (µg/mL)
		0.6	0.59	0.6	0.58	0.6	0.60	1.5	1.50	1.0	1.00	0.99	1.01	0.6	0.59
		1.0	1.00	1.0	0.99	1.0	1.00	2.5	2.48	3.0	2.97	2.97	2.99	1.0	1.01
		4.0	4.00	3.0	3.02	3.0	2.99	3.75	3.78	4.0	3.96	3.97	3.96	1.5	1.50
		6.0	6.00	4.0	4.01	4.0	3.99	5.0	4.97	5.0	4.90	5.05	4.97	2.0	2.00
		8.0	7.98	6.0	6.02	6.0	5.98	6.25	6.262	6.0	6.06	5.99	6.05	2.5	2.49
		10.0	10.01	8.0	7.97	8.0	7.97			8.0	8.03	8.06	8.06		
		12.0	11.99	10.0	9.99	10.0	9.96			10.0	9.90	9.94	9.94		
				12.0	12.01	12.0	12.06								
Mean(X¯)			99.71		99.74		100.13		100.06		99.48	99.91	99.94		99.92
±S. D			0.48		0.91		0.49		0.74		0.90	0.82	0.77		0.69
%RSD			0.49		0.91		0.49		0.74		0.90	0.83	0.77		0.69
% Error			0.18		0.34		0.18		0.33		0.42	0.33	0.31		0.31
				Method of comparison (^11^)											
Mean± S. D	99.19±0.84									100.44±0.86					
t-test		1.262 (2.306)		0.894 (2.306)		2.169 (2.306)		1.685 (2.364)		0.985 (2.306)		0.627 (2.306)	0.075 (2.306)	0.766 (2.446)	
F-test		2.942 (5.143)		1.181 (19.329)		2.826 (5.143)		1.561 (5.786)		1.195 (19.329)		1.273 (19.239)	2.186 (19.239)	1.553 (19.246)	

- Each result is an average of three separate determinations *The values between parentheses are the tabulated t and F values at p = 0.05^29^

**Table 3:** Precision data for the determination of TFH and KTZ by the suggested spectrophotometric techniques.

Drugs	Drug conc. (µg/mL)	Method	Intra-day				Inter-day			
			% found	± SD	% RSD	% Error	% found	± SD	% RSD	% Error
THF	4.0	D ^3^	100.20	0.62	0.62	0.36	99.98	0.22	0.22	0.17
	8.0		100.03	0.25	0.25	0.14	99.98	0.33	0.33	0.19
	10.0		99.60	0.52	0.53	0.30	99.70	0.65	0.65	0.37
	4.0	RD	100.10	0.513	0.51	0.29	99.83	0.72	0.72	0.41
	8.0		100.18	0.246	0.24	0.14	100.00	0.35	0.35	0.20
	10.0		99.50	0.76	0.76	0.44	100.01	0.21	0.21	0.12
	4.0	DD ^1^	100.40	0.83	0.82	0.47	100.00	0.51	0.52	0.30
	8.0		99.90	0.70	0.70	0.40	99.93	0.36	0.36	0.21
	10.0		99.44	0.75	0.75	0.43	99.68	0.62	0.62	0.36
	2.5	IDW	100.20	0.70	0.69	0.40	99.90	0.45	0.45	0.26
	3.75		100.21	0.18	0.18	0.10	99.80	0.49	0.49	0.28
	5.0		99.80	0.40	0.40	0.23	99.70	0.51	0.51	0.30
KTZ	4.0	D ^3^	100.17	0.61	0.61	0.35	100.40	0.51	0.51	0.29
	6.0		100.16	0.28	0.28	0.16	99.98	0.33	0.33	0.19
	8.0		99.46	0.70	0.70	0.40	99.82	0.39	0.39	0.29
	4.0	RD	100.40	0.79	0.79	0.45	100.10	0.29	0.29	0.16
	6.0		100.50	0.80	0.80	0.46	99.85	0.18	0.18	0.10
	8.0		99.36	0.85	0.85	0.49	99.68	0.63	0.63	0.36
	4.0	DD ^1^	100.03	0.55	0.55	0.31	99.83	0.56	0.57	0.32
	6.0		100.48	0.75	0.75	0.43	99.98	0.33	0.33	0.19
	8.0		99.43	0.55	0.55	0.32	99.67	0.61	0.62	0.35
	1.0	DWR	99.83	0.56	0.57	0.32	99.70	0.65	0.65	0.38
	1.5		100.28	0.07	0.07	0.04	100.06	0.36	0.36	0.21
	2.0		99.53	0.61	0.61	0.35	99.60	0.67	0.68	0.39

*N. B.* Each outcome is the mean of three independent assessments

#### Selectivity

The proposed techniques were employed to estimate TFH and KTZ in their prepared co-formulated tablet. The suggested spectrophotometric techniques measured TFH and KTZ without any excipient influence. The outcomes in Table 4 demonstrate the suggested approaches’ high selectivity.

**Table 4:** Assay findings for TFH and KTZ determination in a prepared tablet utilizing the suggested spectrophotometric techniques.

	^3^D		RD		DD^1^		IDW		^3^D		RD		DD^1^		DWR	
Mix No	Taken Conc. (µg/mL)	%Found	Taken Conc. (µg/mL)	%Found	Taken Conc. (µg/mL)	%Found	Taken Conc. (µg/mL)	%Found	Taken Conc. (µg/mL)	%Found	Taken Conc. (µg/mL)	%Found	Taken Conc. (µg/mL)	%Found	Taken Conc. (µg/mL)	%Found
	THF								KTZ							
1	2.5	99.65	2.5	99.50	2.5	98.70	1.5	99.01	1.0	99.60	1.0	99.9	1.0	99.8	0.6	99.65
2	5	101.01	5	100.00	5	100.00	2.5	98.92	2.0	99.30	2.0	100.20	2.0	101.00	1.0	101.01
3	7.5	100.01	7.5	100.01	7.5	99.90	3.75	100.01	3.0	100.40	3.0	98.80	3.0	100.01	1.5	100.01
Mean	100.22		99.84		99.53			99.31	99.77		99.63		100.27			100.20
± S.D.	0.70		0.29		0.72			0.70	0.56		0.73		0.64			0.70
%RSD	0.70		0.29		0.72			0.60	0.57		0.74		0.63			0.70
% Error	0.40		0.16		0.41			0.40	0.32		0.42		0.36			0.40

N. B. Values between parentheses are the tabulated t and F values at p = 0.05^29^

### Applications

#### Analyzing the prepared tablet

The produced tablets’ varied TFH and KTZ ratios were examined using the suggested spectrophotometric techniques. The results shown in Table 4 showed that these approaches were accurate, since they closely matched the results from the reported chromatographic method^11^. No discernible variations in precision and accuracy were found between the two approaches according to statistical analysis, which included the variance ratio F-test and Student’s t-test^29^.

#### Evaluation of greenness of the proposed methods

**Fig. 7:**
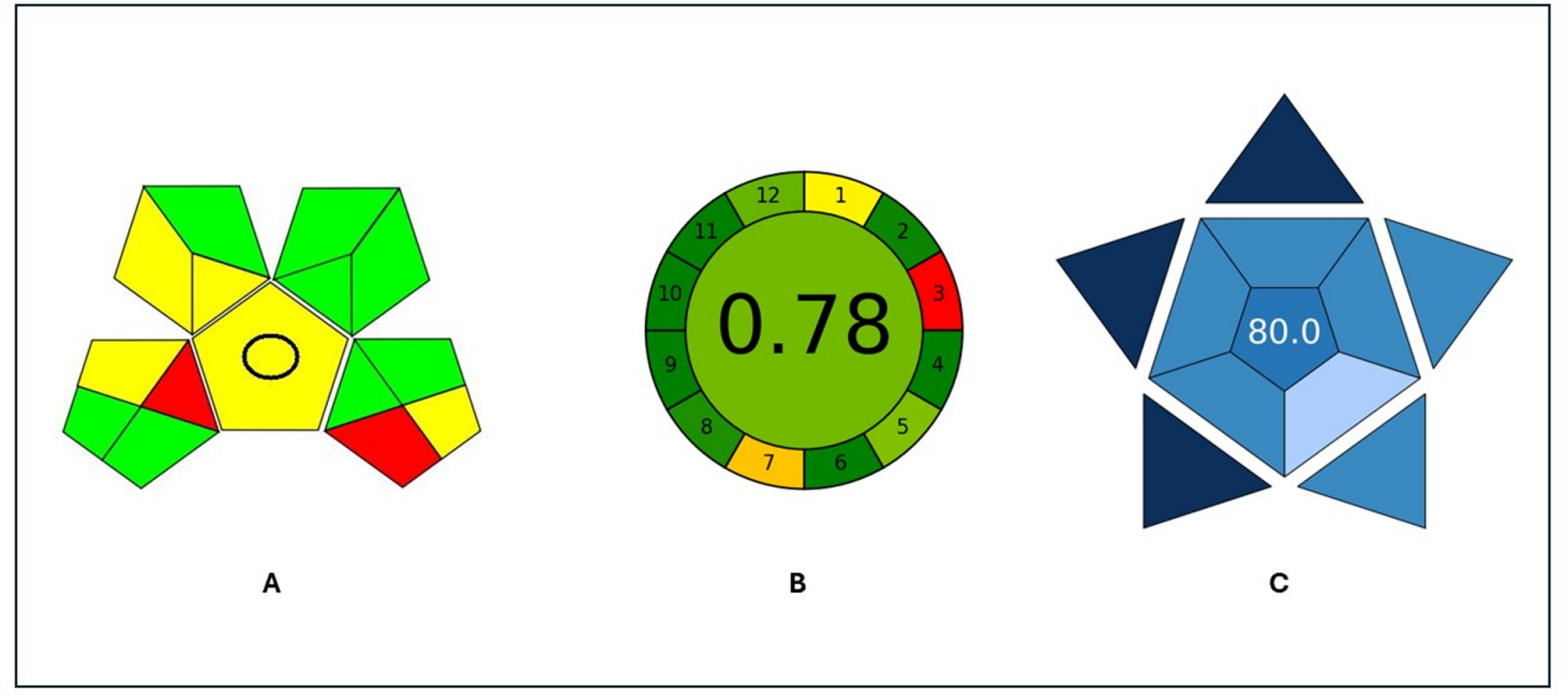
Evaluation of the greenness profile and blueness of the proposed methods using GAPI (**A**), AGREE (**B**) and BAGI (**C**).

Protecting people and the environment from solvents and organic hazards resulting from chemical and pharmaceutical compounds is a critical function of analysts. To evaluate the ‘greenness’ of analytical methods, many metrics are followed, such as the Analytical Eco-scale score^30^, GAPI^31^ and AGREE^32^. The result of an Analytical Eco-scale assessment is the number that is subtracted from 100 (ideal green analysis) by penalty points awarded. These points show the hazards used during the analytical process. The higher the value, the greener the analysis will be. The suggested methods’ Eco-scale score of 97 indicated that they were a very good green approach (Table 5). GAPI which is color color-coded scoring tool that uses the colors (green, red, yellow) which allows quick assessments of the greenness of the method^33,34^. (Fig. 7**)**, and the AGREE calculator that is based on the significant mnemonic, which encapsulates the 12 principles of green analytical chemistry. These principles guide the evaluation of analytical methodologies to ensure they are environmentally benign and safer for human health^35–37^. (Fig. 7).

**Table 5:** Analytical Eco-scale Penalty point calculations for the suggested approaches.

Reagents	Penalty points
Technique spectrophotometry (Less than 0.1 kWh per sample)	0
Solvent (water)	0
Waste	3
Occupational hazard	0
Total penalty points	3
Score *	97

* If the score is more than 75, it shows excellent green analysis. If the score is more than 50, it shows an acceptable green analysis. If the score is less than 50, it shows deficient green analysis

#### Evaluation of blueness of the proposed methods

Within the scope of white analytical chemistry (WAC), the Blue Applicability Grade Index (BAGI) was unveiled in 2023 as a new measure for evaluating the applicability of analytical techniques and as an adjunct to current green metrics^38^.

BAGI focuses on 10 essential characteristics that are crucial to the method application, in contrast to conventional green metrics that emphasize environmental effect. These include the type of analysis, the capacity to identify multiple analytes at once, the necessary analytical methods and equipment, the quantity of samples that can be treated concurrently, sample preparation, hourly sample throughput, reagent and material selection, the need for preconcentration, the level of automation, and the required sample size^39^.

The overall evaluation is shown as a pictogram of an asteroid with a number in the middle. The pictogram’s color gradient shows how well the approach satisfies the established criteria: white indicates no compliance, light blue recommends poor compliance, blue indicates medium compliance, and dark blue indicates high compliance. The overall analytical method score, which ranges from 25 to 100, is represented by the number inside the BAGI pictogram. The lowest level of applicability is represented by a score of 25, while the top performance is indicated by a score of 100. For the analytical process to be considered realistic, it is recommended that the final score be higher than 60^38^(Fig. 7).

By improving knowledge of method practicality, BAGI hopes to become recognized as a useful instrument for method evaluation in the chemical world.

### Comparison between the proposed methods

To provide a clearer evaluation of the proposed spectrophotometric methods, a comparative analysis was conducted. Table 6 summarizes the main advantages and disadvantages of each method in terms of sensitivity, simplicity, cost-effectiveness, and applicability to pharmaceutical dosage forms. This comparison aims to assist the selection of the most suitable approach based on specific analytical requirements.

**Table 6:** The advantages and disadvantages of each proposed spectrophotometric method.

Method	Advantages	Disadvantages
I. Third derivative spectrophotometry^40–42^	- Time saving, cheap, simple, more environmentally friendly, shows reliability, precision, accuracy and could be used for routine analysis of the cited drugs.	- Strong dependence on instrumental parameters and its tendency to amplify noise, leading to distorted spectra.
II. Ratio difference method^43,44^	- Simplicity, accuracy and reproducibility - The ability to solve severely overlapped spectra without prior separation, meanwhile, it doesn’t require any sophisticated apparatus or computer programs.	- Requires appropriate divisor spectrum that should compromise between minimal noise and maximum sensitivity
III. First derivative of ratio spectrophotometric method^43^	- Good selectivity for components with overlapping spectra - Useful in multicomponent analysis	- The multiple manipulating steps: division, then calculating the derivative.
IV. Induced dual wavelength method^25,45^	- It is good for application to a binary mixture having completely overlapped zero-order absorption spectra -Need no divisor.	- Requires precise two wavelength selection - Need calculation of the equality factor (F).
V. Dual wavelength resolution technique^45^	- Recovering the original spectra of the cited drugs which act as the spectral profile of them. -Enhances the sensitivity of the results using measurements at peak maxima.	-Must be used complementary to another spectrophotometric method such as Induced Dual Wavelength Method.

## Conclusion

Five straightforward, precise spectrophotometric techniques for the simultaneous determination of TFH and KTZ were presented in this work. These techniques included DWR, IDW, DD^1^, RD, and D^3^. When the assay findings from these techniques were contrasted to those from a published HPLC method, excellent agreement was found. For TFH and KTZ bespoke routine analysis in their co-formulated pharmaceutical product, the proposed procedures are suitable. The univariate approaches of the proposed methods need less mathematical processing and provide greater linearity ranges for both medications than the published chromatographic method. These advantages make the proposed methods superior to the latter.

## Data Availability

The datasets generated and/or analyzed during the current study are included in this submitted article.
